# A157 ASSOCIATION OF SUBMUCOSAL INVASION WITH THE PARIS CLASSIFICATION OF POLYPS IN THE LOWER GASTROINTESTINAL TRACT

**DOI:** 10.1093/jcag/gwad061.157

**Published:** 2024-02-14

**Authors:** M Scaffidi, R Bechara

**Affiliations:** Medicine, Queen's University Faculty of Health Sciences, Kingston, ON, Canada; Medicine, Queen's University Faculty of Health Sciences, Kingston, ON, Canada

## Abstract

**Background:**

Endoscopic evaluation of the colorectum allows for diagnosis of colorectal cancer (CRC) and advanced polyps. In particular, polyps can be described in terms of gross morphology using the Paris classification. Previous work has demonstrated that submucosally invasive (SMI) cancer may correlate with the Paris classification subtypes.

**Aims:**

To determine whether the Paris classification is associated with SMI in a Canadian setting.

**Methods:**

We conducted a retrospective single-centre study using data from 2016 to 2023 of patients with colorectal lesions that were referred for endoscopic submucosal dissection (ESD). Using the Paris classification, we classified polyps as either protruding type (0-I) or flat type (0-IIa, 0-IIb, 0-IIc, 0-III) lesions. Protruding type lesions were further classified as sessile (0-Is), pedunculated (0-Ip) and pseudo-pedunculated (0-Isp) subtypes. Mixed lesions, with two concomitant subtypes, were categorized using the higher classification subtype. We categorized polyps as: protruded lesions, including any mixed lesions with a flat lesion; isolated flat elevated lesions; and flat lesions, both mixed and isolated lesions. We also recorded polyp location, prior endoscopic mucosal resection (EMR), previous biopsy, and polyp diameter (in cm), age, sex, and American Society of Anesthesiology (ASA) class. The primary outcome was SMI as identifed on histopathology. We used logistic regression with backward stepwise entry, Fisher exact test with relative risk (RR) and 95% confidence interval (95% CI), and independent samples t-test. Statistical tests were two-tailed and significant at Pampersand:003C0.05.

**Results:**

In our sample of 130 patients, the mean age was 69.2 years (standard deviation [SD] 10.4), most were male (*n*=78, 60%), and typically had an ASA score of 3 (*n*=77, 59.2%). Most polyps occured in the rectum (*n=*65, 50%) with a mean length was 4.5 cm (SD 2.2 cm). The most common subtype was IIa (*n*=59, 45.4%). Starting with the covariates of age, sex, ASA, prior EMR, prior biopsy at the same, and length of polyp, the logistic regression model reduced them to three: prior EMR, prior biopsy at the same site, and length. Univariate analysis of these three variables found that there was no significant difference with polyp length and SMI (P=0.147) and no significant association with prior EMR on SMI (RR=0.8 [95% CI 0.3 to 1.9]. The presence of prior biopsies at the same site of the lesions was significantly associated with SMI (RR=2.4 [95% CI 1.2 to 4.7])

**Conclusions:**

We did not find that the Paris classification for type of polyp lesion did not correlate with the presence of submucosal invasion, nor did prior EMR, age, sex, or length of polyp. The presence of a prior biopsy, however, did in fact tend to predict submucosal invasion. Future work should aim to incorporate mortality data and response to treatment into the above analysis.

**Funding Agencies:**

None

